# Comprehensive analysis of the ceRNA network in coronary artery disease

**DOI:** 10.1038/s41598-021-03688-9

**Published:** 2021-12-20

**Authors:** Weikang Bian, Xiao-Xin Jiang, Zhicheng Wang, Yan-Rong Zhu, Hongsong Zhang, Xiaobo Li, Zhizhong Liu, Jing Xiong, Dai-Min Zhang

**Affiliations:** 1 Department of Cardiology, Nanjing First Hospital, Nanjing Medical University, 68 Changle Road, Nanjing, 210006 Jiangsu People’s Republic of China; 2 Department of Pharmacology, China Pharmaceutical University, Nanjing, 210009 Jiangsu People’s Republic of China

**Keywords:** Cardiovascular biology, Cardiovascular diseases

## Abstract

With the rapid aging of the population, coronary artery disease (CAD) has become one of the most fatal chronic diseases. However, the genetic mechanism of CAD is still unclear. The purpose of this study is to construct the lncRNA-miRNA-mRNA regulatory network for CAD diseases and systematically identify differentially expressed genes in patients with coronary heart disease. In this study, two lncRNA datasets (GSE69587 and GSE113079) and a microRNA dataset (GSE105449) which contained 393 and 38 CAD samples were selected. In addition, two mRNA datasets which named GSE113079 (98 CAD samples) and GSE9820 (8 CAD samples) were selected to search the differentially expressed genes (DEGs). By comparing the expression data between CAD and control samples, a total of 1111 lncRNAs, 2595 mRNAs and 22 miRNAs were identified. Based on the DEGs, a lncRNA-miRNA-mRNA ceRNA network was constructed to explore the hub nodes in CAD. In the ceRNA network, the lncRNAs KCNQ1OT1 and H19 showed high connectivity with the nine miRNAs. GO and KEGG results showed that genes in ceRNA networks were mainly involved in nitrogen compound metabolic process, PI3K-Akt signaling pathway and retrograde endocannabinoid signaling. These findings will improve the understanding of the occurrence and development mechanism of CAD.

## Introduction

Coronary artery disease (CAD) is one of the most important diseases threatening human life and health around the world^1^. Recent studies have confirmed that coronary heart disease (CHD, also known as CAD) is an inflammatory disease involving the interaction of multiple factors, such as immunity, environment and heredity, and its course can be aggravated by factors such as hemodynamic changes, infection, stress and inflammatory response^2^. Due to the relatively complex risk factors and pathogenesis of CHD, such as ethnic differences and genetic heterogeneity, research on the underlying pathogenesis and the mutual influence of CHD has been carried out for early diagnosis and prevention of this disease. However, the genetic basis of the pathogenesis of CHD is still unclear.

microRNAs (miRNAs) are highly conserved endogenous small-molecule single-stranded noncoding RNAs containing 20–24 oligonucleotides^3^. miRNAs are characterized by tissue- and stage-specific expression and have been relatively well conserved in species over the course of evolution^4^. They exist in almost all eukaryotic microorganisms. miRNAs bind to noncoding regions of target genes through base pairing to promote degradation of target genes or inhibit posttranscriptional translation^5^. The expression of approximately 1/3 of the genes in the human genome is regulated by miRNAs and is involved in human growth and development, organ formation, cell proliferation and apoptosis, fat metabolism and other life processes^6^. Some studies have shown that miRNA-1, miRNA-126, miRNA-133, miRNA-208 and miRNA-499 have abnormal expression levels in patients with CHD, suggesting that these miRNAs may be potential biomarkers of CHD, but no definitive conclusion has been reached^7^.

Long noncoding RNA (lncRNA) is a kind of noncoding RNA molecule with a transcript length greater than 200 nt; this type of RNA is less well conserved and species specific^8^. Several studies have demonstrated that lncRNAs are involved in biological processes such as cell transduction, chromosome modification, transcription and translation regulation and the occurrence of a variety of diseases^9^. Two lncRNAs named ENST00000444488.1 and uc010yfd.1 were identified as novel lncRNA biomarkers for diagnosing CAD by a transcriptome-wide overview of aberrantly expressed lncRNAs in CAD patients^10^. Myocardial infarction-associated transcript (MIAT) is the earliest recognized lncRNA with risk factors^11^. A single SNP locus can change the expression of MIAT and increase susceptibility to acute myocardial infarction^12^. Genome-wide association analysis of CAD showed that the 9p21 region of the chromosome was the most susceptible to CAD infection^13^. This region contains a functional lncRNA antisense non-coding RNA in the INK4 locus (ANRIL) block that has a direct regulatory role in cardiovascular diseases^13^. Therefore, it is necessary to systematically identify differentially expressed lncRNAs in patients with CAD and to explain how lncRNAs participate in the regulation of the occurrence and development of CAD.

In this study, our goal was to construct the lncRNA-miRNA-mRNA regulatory network for CAD diseases and systematically identify differentially expressed genes in patients with CHD to screen potential functional genes. This study will provide basic data regarding the pathogenesis and treatment of CAD.

## Materials and methods

### Data sources

**Figure 1:**
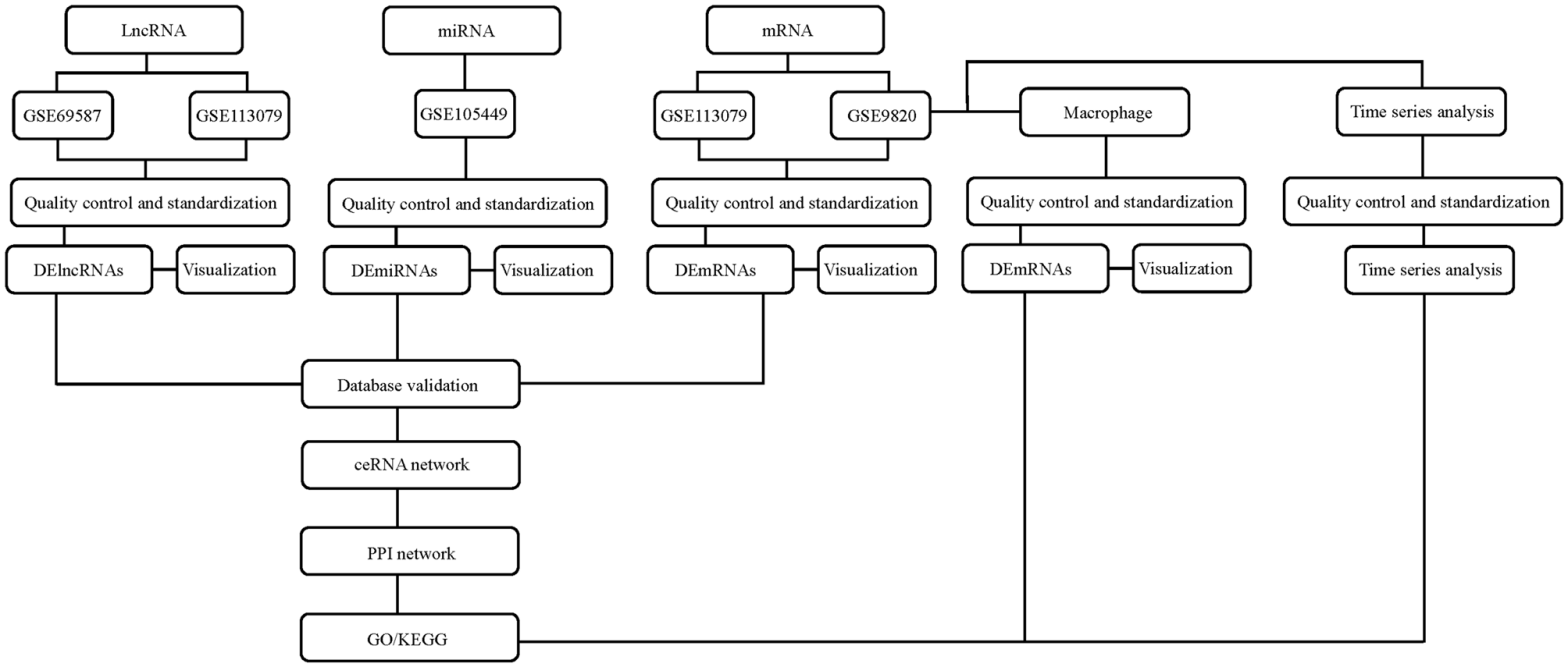
The flow chart of the methodology of work.

The Gene Expression Omnibus^14^ (GEO, http://www.ncbi.nlm.nih.gov/geo) database was used to search the datasets that contained the CAD samples. Two lncRNA datasets, namely, GSE69587 and GSE113079, were used in this study. GSE69587 contained 3 control samples and 3 CAD samples (Fig. 1). GSE113079 contained 93 CAD samples and 48 heathy control samples. Both datasets contained microarray data. The miRNA dataset GSE105449, which included 38 CAD samples and 42 control samples, was also analyzed. Two mRNA datasets, named GSE113079 and GSE9820, were selected for further analysis. The GSE113079 dataset contained 98 CAD samples and 48 healthy control samples. For the GSE9820 dataset, 13 resting monocyte healthy control samples and 8 resting monocyte CAD samples were selected for expression analysis. Meanwhile, 15 control samples and 19 CAD samples from GSE9820 were selected for gene expression analysis in macrophages. All the samples used in this study were the circulating monocytes which were isolated from peripheral blood samples. The expression data were transformed by log2 and standardized to obtain a series matrix file.

### Differential expression analysis

Before analyzing the differences in expression, the probe was annotated. If multiple probes corresponded to the same gene, the average value for the multiple probes was taken as the expression value of the gene. The R package limma^15^ was used to perform the differential expression analysis of genes between the CAD groups and the control groups using the microarray data. The screening threshold for significant differences in mRNA, miRNA and LncRNA expression was *p* < 0.05 and |log2FC|> 0.585 (i.e., FC > 1.5 and FC < 1/1.5). After identifying the significantly different lncRNAs of GSE69587 and GSE113079, combination of the different lncRNAs was performed to obtain the final set of significantly different lncRNAs for downstream analysis. Similarly, the significantly different mRNAs were also determined by combination of the significantly different genes in the two data sets.

### Construction of the ceRNA network

After significant differences in lncRNAs, miRNAs and mRNAs were obtained, the miRMap^16^, miRanda^17^, miRDB^18^, TargetScan^19^ and mirTarBase^20^ databases were used to construct the miRNA and mRNA interaction network. To further improve the reliability of predicted miRNA-mRNA interactions, the miRNA-mRNA interaction relationships need to be present in at least 3 databases before they are retained. The interaction network of differentially abundant miRNAs and lncRNAs was constructed based on the interaction information of miRNA-targeted lncRNAs in the StarBase database^21^. Based on the above two interaction networks, the interaction information consistent with the expression regulation trend of the competing endogenous RNA (ceRNA) network (lncRNA negatively regulates miRNA, and miRNA negatively regulates mRNA) was screened out to obtain the ceRNA regulatory network. Finally, the control network was visualized with cytoscape^22^.

### PPI network analysis

The significantly different mRNAs in the ceRNA network were analyzed by using the STRING^23^ (https://string-db.org/) online database for protein–protein interaction (PPI) analysis. In this analysis, the confidence of protein interactions was set to high, i.e., only interactions with a combined protein score of ≥ 0.7 were retained. After obtaining the PPI network, the network was visualized and analyzed using cytoscape software. For larger interaction networks, the hub gene in the PPI network can be obtained by analyzing the degree of connectivity of nodes using the cytoHubba^24^ plug-in based on network statistics.

### Gene function enrichment analysis

Based on the Gene Ontology database^25^ and KEGG pathway database^26^, functional enrichment analysis was performed using the genes in ceRNA network, time series groups and macrophage, respectively. Using a statistical algorithm (Fisher's exact test) to determine the set of genes and identify which item/channel correlation was the largest, the results of the analysis of each entry were assigned a statistical value (p) representing the significance. The smaller the value is, the more likely the entry/pathway, indicating the correlation. That is, the greater the difference in the expression level is, the more likely that the gene associated with the listed entry/pathway influences cellular life activities and warrants further research.

### Time series analysis

The R package Mfuzz^27^ was used for time series analysis of mRNA expression data. The probe was annotated before time series analysis. If multiple probes corresponded to the same gene, the average value of the multiple probes was taken as the expression value of the gene. After the expression matrix was standardized, normalization was carried out to ensure that the expression levels of different genes were in the same range. Then, the standard deviation (SD) ≥ 0.05 genes were screened by standard deviation for clustering (if the SD was too small, the gene was considered to have almost no change in expression and was not included in time series analysis). The KNNW method was used to cluster the expression data of multiple time points, and the genes with similar expression trends were grouped into one class. According to the number of time points and clustering results, the final cluster number could be adjusted continuously so that it not only reflected the downward trend but also achieved the purpose of gene screening.

### Consent for publication

All authors approved its publication.

## Results

### Differentially expressed mRNAs, miRNAs and lncRNAs

**Figure 2:**
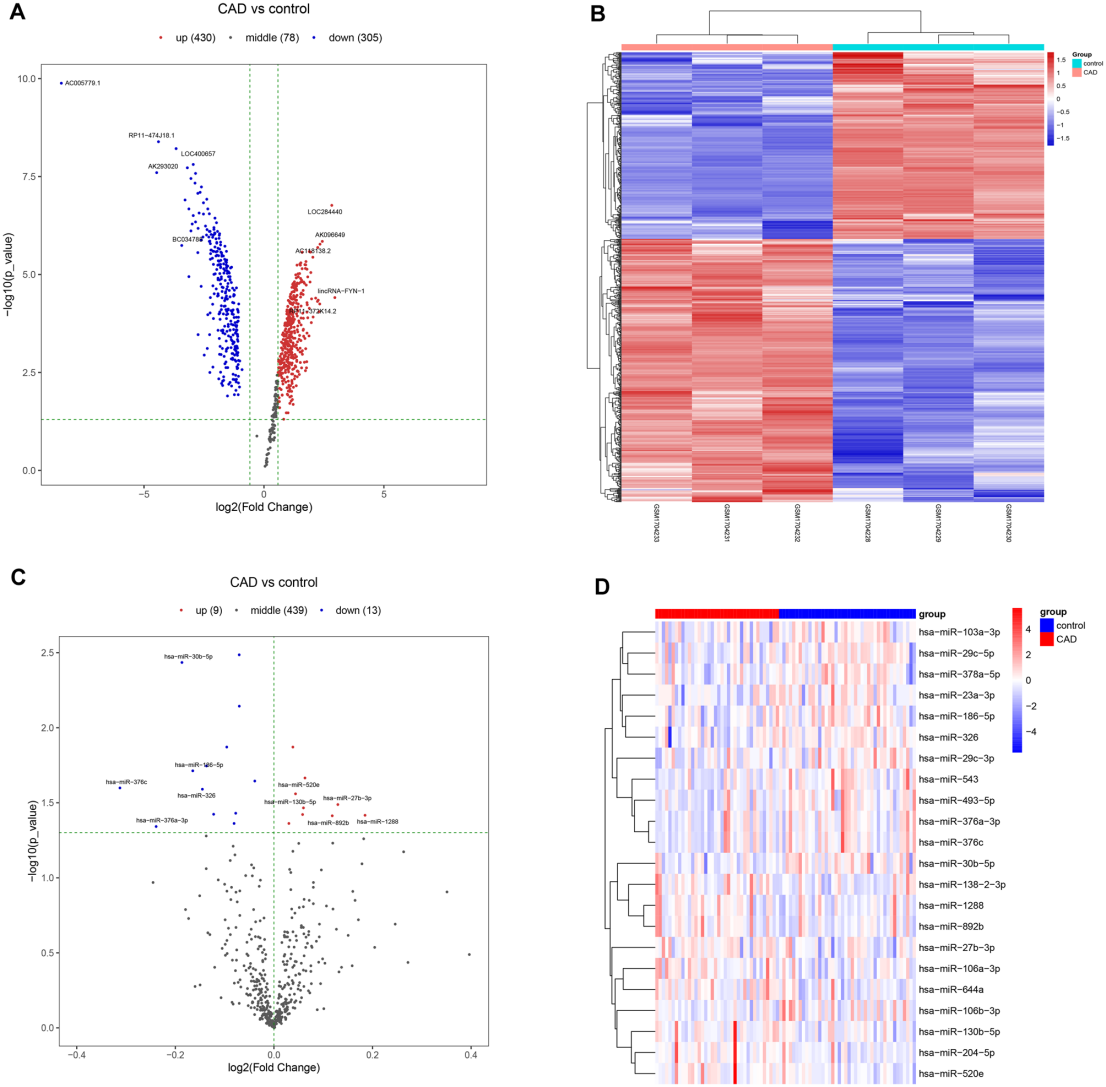
Volcano and heatmap of the lncRNA (GSE69587) and miRNAs (GSE105449). (**A**, **C**) were the volcano map in which downregulated genes were represented as blue points and upregulated genes were represented as red points. (**B**, **D**) were the heatmaps of lncRNAs and miRNAs in which the downregulated gene is represented by the blue rectangles and upregulated gene is represented by the red rectangles. The screening threshold for significant differences in mRNA, miRNA and lncRNA expression was *p* < 0.05 and |log2FC| > 0.585 (i.e., FC > 1.5 or FC < 1/1.5).

By comparing the CAD groups with the control groups, we identified 1111 lncRNAs, 2595 mRNAs and 22 miRNAs which showed the significantly expressions (Table 1, Fig. 2). Among the 1111 significantly expressed lncRNAs, 499 showed downregulated expression, and 612 showed upregulated expression. In total, 1238 significantly expressed mRNAs showed downregulated expression, and 1357 mRNAs showed upregulated expression. The 22 significantly expressed miRNAs mainly contained 13 downregulated miRNAs and 9 upregulated miRNAs. Then, differentially expressed genes from the two datasets were intersected to obtain the common genes. Seven genes, namely, CCL3L3, KIR2DL4, IL8, GNLY, CXCL2, C1orf150, and CCL3, showed downregulated expression in both datasets. Only one lncRNA, named H19, was identified in both datasets.

**Table 1:** Statistics of the differentially expressed genes.

Datasets	Down-regulation	Up-regulation	Total
GSE69587	305	430	735
GSE113079	193	183	376
All_lnc	499	612	1111
GSE113079	1139	1347	2486
GSE9820	106	10	116
All_mRNA	1238	1357	2595
GSE105449	13	9	22
Macrophage	58	94	152

### Construction of the ceRNA network

**Figure 3:**
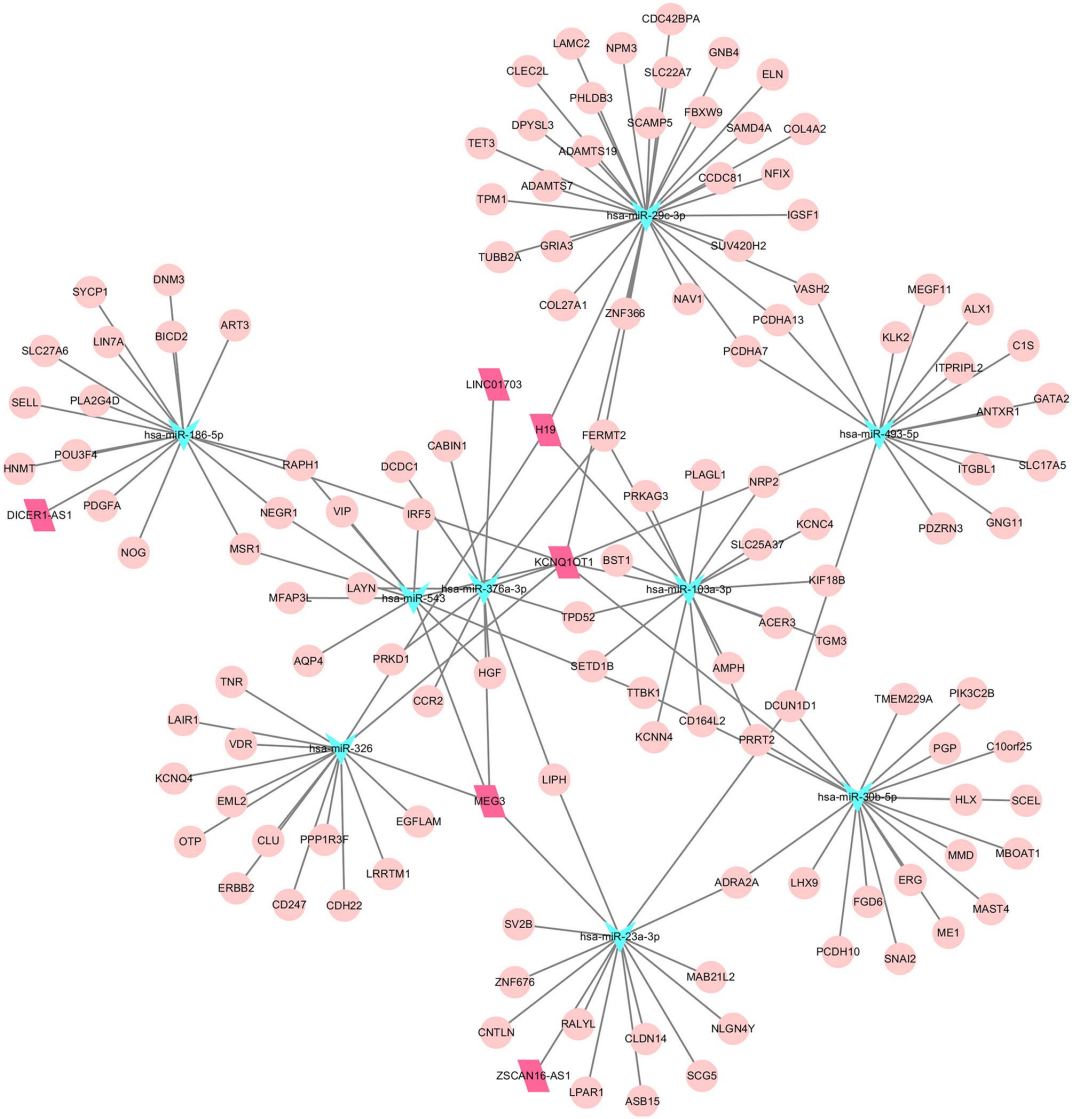
ceRNA network based on the upregulated lncRNAs. The shape represents the molecular type, the circle represents the mRNA, the arrow triangle represents the miRNA, and the rhomboid represents the lncRNA. The color represents upregulated expression, cyan represents downregulated expression, and red represents upregulated expression.

We identified 22 miRNAs that were differentially expressed between the CAD groups and the healthy group. Six miRNA target gene databases were utilized to select the interactions of miRNAs and to construct the regulatory lncRNA-miRNA-mRNA interaction network. Finally, two ceRNA networks were constructed based on 9 downregulated miRNAs and 2 upregulated miRNAs. As Fig. 3 shows, the upregulated lncRNA KCNQ1OT1 showed regulatory relationships with 8 miRNAs, excluding has-miR-23a-3p. MEG3 also showed connectivity with four miRNAs, namely, has-miR-23a-3p, has-miR-326, has-miR-543 and has-miR-376a-3p. For miRNAs, has-miR-376a-3p showed the most interactions with lncRNAs, such as LINC01703, KCNQ1OT1 and MEG3. In addition, interactions between lncRNAs and 2 upregulated miRNAs were also selected and used to construct the ceRNA networks. For networks based on the 2 upregulated miRNAs, has-miR-204-5p and has-miR-27b-3p were connected indirectly by common regulatory genes such as MAPK9, FOSB, ASPH and FAM126A. Among the 2 miRNAs, has-miR-27b-3p was regulated by 4 lncRNAs, namely, RMST, TRIM52-AS1, AC005779.1 and AL627309.3. Three lncRNAs, namely, LINC01977, LINC00641 and SP2-AS1, showed regulatory relationships with has-miR-204-5p.

### GO and KEGG pathway analyses of mRNAs in ceRNA networks

**Figure 4:**
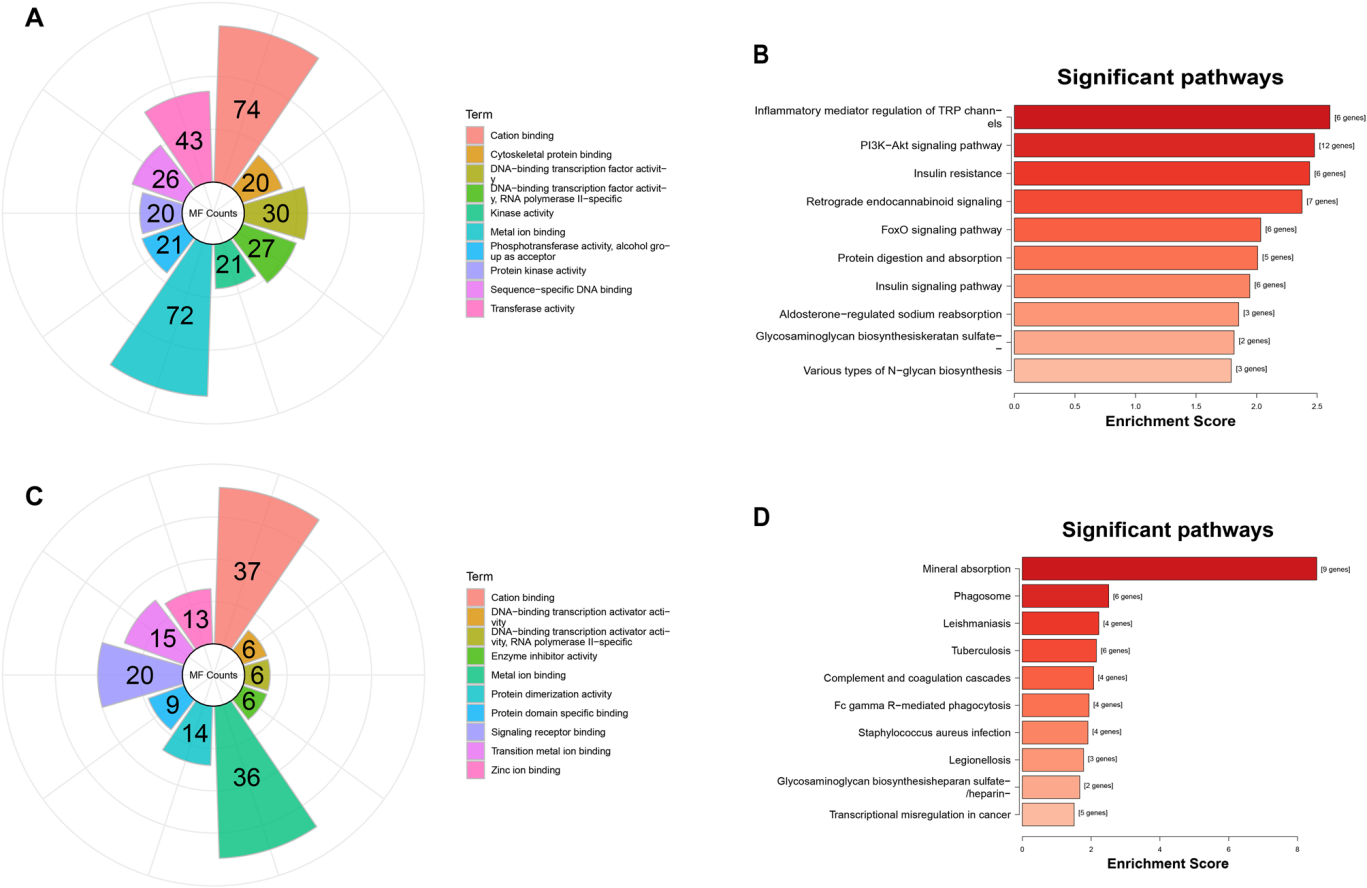
GO and KEGG analyses of the genes in ceRNA networks (**A**, **B**) and macrophages (**C**, **D**). (**A**, **C**) were the molecular function terms in which the area of the sector corresponds to the number of terms. (**B**, **D**) were the KEGG pathways results in which the X-axis is enrichment score and the Y-axis is the KEGG pathways terms.

To understand the functions of the differentially expressed mRNAs involved in the 2 ceRNA networks, a total of 241 genes were selected to perform GO and KEGG pathway analyses. The top 10 GO terms that could be classified into molecular function (MF), biological process (BP) and cellular component (CC) among the 241 genes are listed in Fig. 4. The main biological processes that the genes were involved in were nitrogen compound metabolic process, macromolecule metabolic process, anatomical structure development and regulation of nitrogen compound metabolic process. The significantly enriched KEGG pathways^28^ of the mRNAs were mainly inflammatory mediator regulation of TRP channels, PI3K-Akt signaling pathway and retrograde endocannabinoid signaling. Regarding the PI3K-Akt signaling pathway, several upregulated genes, such as HGF, ERBB2, IL7R and COL4A2, were involved in this pathway (Fig. S1).

### PPI network analysis

**Figure 5:**
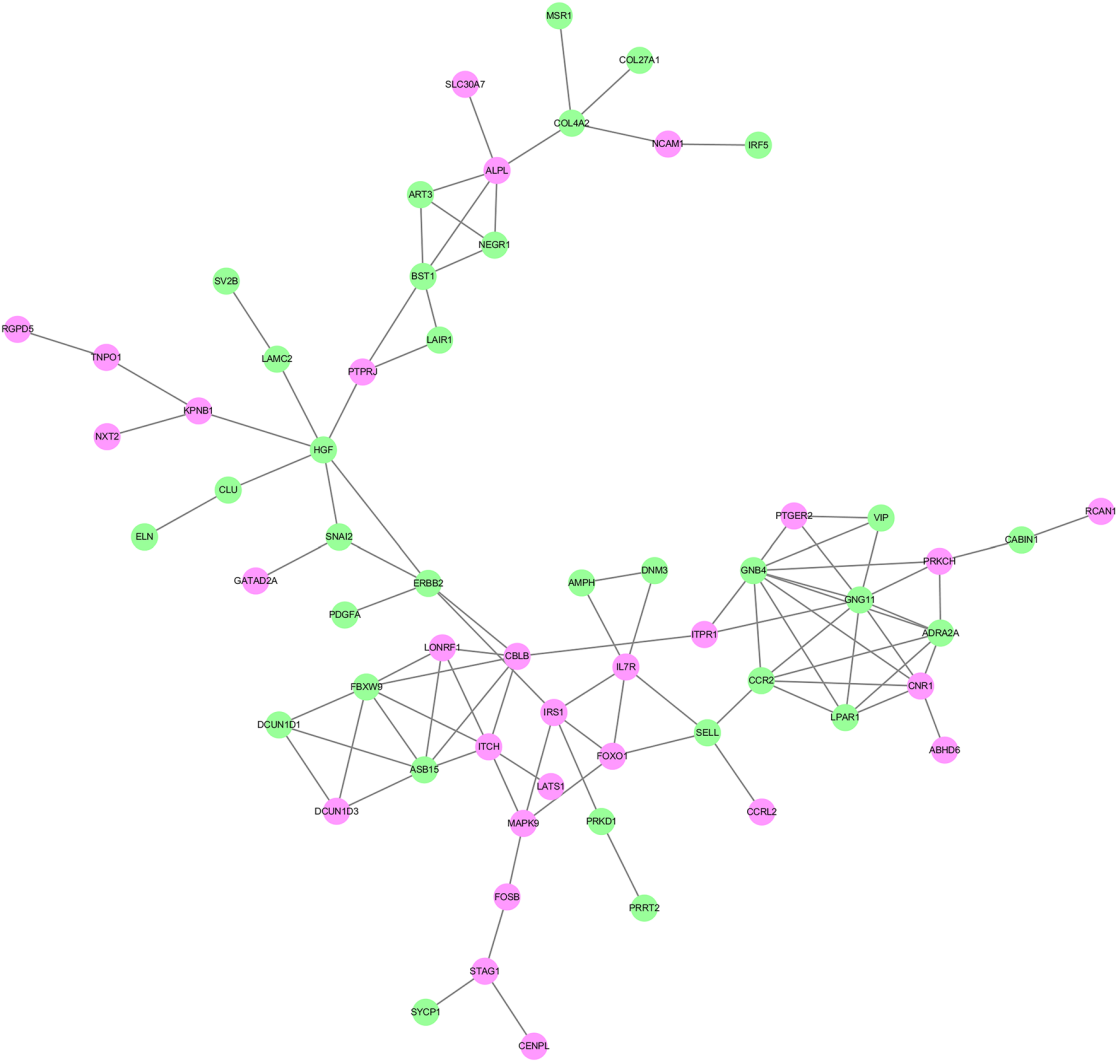
PPI network of the genes in ceRNA networks. Purple circles indicate upregulated expression and green circles indicates downregulated expression. The confidence of protein interactions was set to high. Only the combined protein score which was equal or greater than 0.7 was retained.

The mRNAs involved in the ceRNA networks were selected to perform the PPI network analysis based on the STRING database. As Fig. 5 shows, the PPI networks with a high degree of confidence (combined score > 0.7) contained 84 nodes and 110 edges. Then, cytoscape was used to analyze the connectivity of the 84 nodes. Two genes, namely, GNB4 and GNG11, showed the highest degree of connectivity, both with a value of 9. Eight genes exhibited the second highest degree of connectivity, namely, FBXW9, ASB15, HGF, CCR2, CBLB, ITCH, ADRA2A and CNR1.

### Time series analysis of the differentially expressed genes

**Figure 6:**
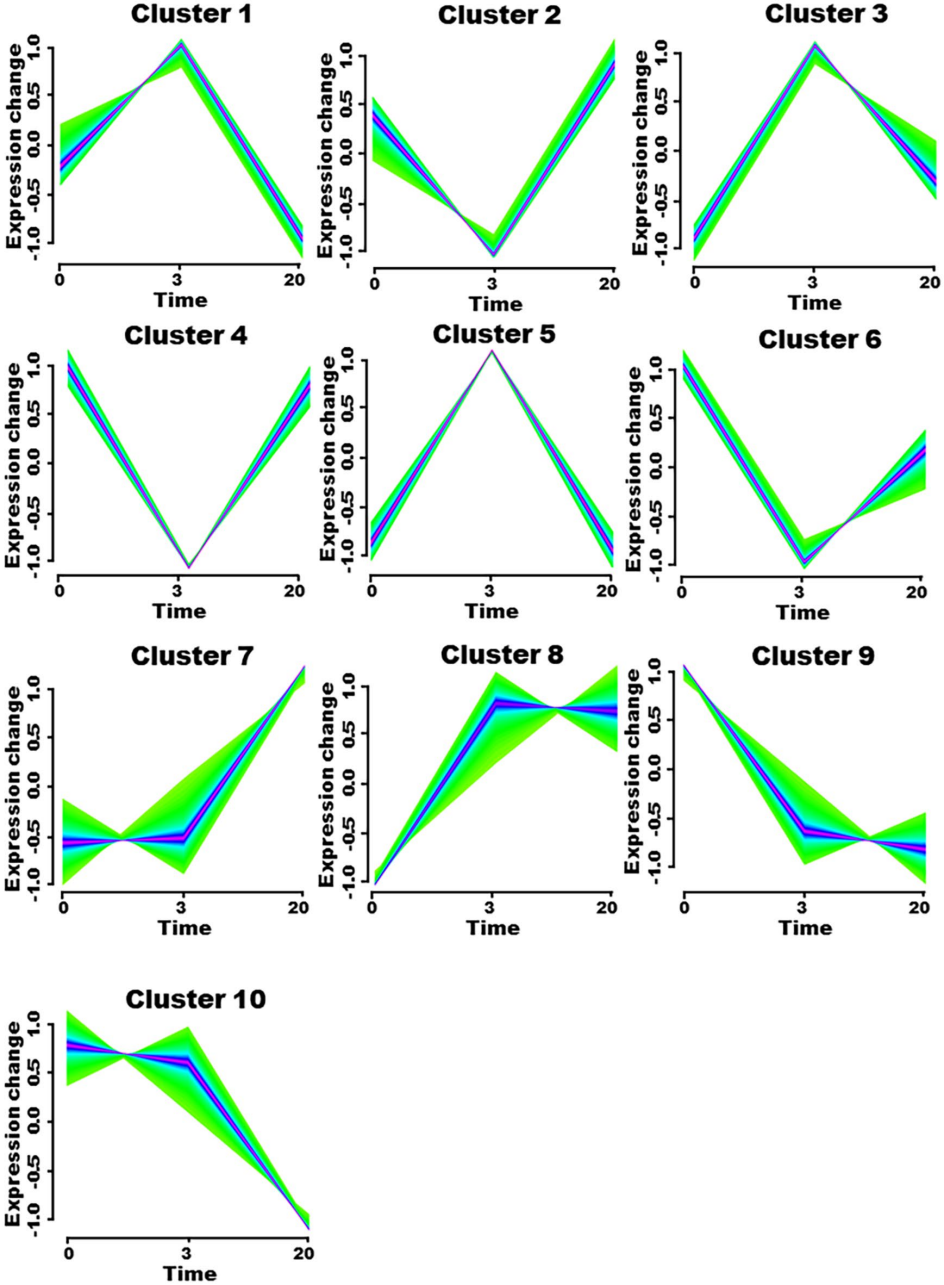
Time series analysis. Cluster 1, 3 and 5 were genes whose expression go up first and then down. Cluster 2, 4 and 6 were genes whose expression go down first and then up. Clusters 7 and 8 were consistently upregulated genes. Clusters 9 and 10 were consistently downregulated genes. The X-axis is time series, and the Y-axis is gene expression.

To understand the dynamic changes in the genes involved in CAD, expression data from 31 0-h resting monocyte samples, 31 3-h stimulated monocyte samples and 34 20-h macrophage samples were extracted from the GSE9820 dataset. Using the R package Mfuzz, 10 clusters were obtained that showed the different expression trends of the genes in the CAD samples (Fig. 6). Among the 10 clusters, cluster 1, cluster 3 and cluster 5 mainly contained the genes that showed the early increase and late decrease expression trend. However, cluster 2, cluster 4 and cluster 6 showed the reverse trend. The expression of genes in cluster 7 and cluster 8 was continuously upregulated. Genes that showed a downward trend in expression were classified into cluster 9 and cluster 10. Then, the intersection of genes between the ceRNA gene set and clustered gene set was further analyzed. A total of 11 genes of the ceRNA networks showed a continuous decline in expression, namely, LMNB1, NFIX, SELL, PCDHA13, FAM76B, VIP, KLK2, PCDHGA5, MAB21L2, MFAP3L and COL27A1. Nine genes of the ceRNA networks showed continuous increases in expression, namely, KCNJ1, G3BP2, KCNC4, CRIM1, UBE2V2, BNC2, PRRT2, SLC17A5 and DPYSL3.

### Analysis of macrophage expression

To investigate the expression of genes in macrophages, 15 control samples and 19 CAD samples from GSE9820 were extracted for further analysis. A total of 152 significantly expressed genes were found in macrophages, which included 94 upregulated and 58 downregulated genes. Among the 94 up-regulated genes in macrophages, 8 genes were also identified in the continuously up-regulated group of the time series analysis, containing HSPA1B, GPR92, TGM2, MT1JP, SPRED1, HS3ST1, MT1H and MT1M. Two genes, CD302 and CSF3R, were found down-regulated both in macrophages and time series analysis. GO and KEGG pathway analyses were also performed based on the differentially expressed genes of macrophages. GO terms were mainly enriched in cation binding, metal ion binding and signaling receptor binding. The KEGG pathways were mainly enriched in mineral absorption, phagosome, leishmaniasis and tuberculosis.

## Discussion

According to accumulating evidences, miRNAs and lncRNAs play important roles in human disease occurrence and development^29^. Although the pathogenesis is unclear, an increasing number of researchers have paid attention to the functions of ceRNA networks in which lncRNAs could indirectly affect the expression of mRNAs by interacting with miRNAs. Several ceRNA expression networks have been reported in human tumors, such as hepatocellular carcinoma, carcinoma of the uterine cervix and polycystic ovarian syndrome^30^.

In this study, we identified 111 lncRNAs, 2595 mRNAs and 22 miRNAs that were differentially expressed in CAD samples based on the NCBI database. Subsequently, 5 miRNA target mRNA databases and 1 lncRNA target mRNA database were used to construct the ceRNA networks. Using GO and KEGG pathway analyses, the functions and signaling pathways of the mRNAs in the ceRNA network were confirmed. We further analyzed the PPIs based on the mRNAs involved in the ceRNA networks.

By comparing the two lncRNA datasets, only one lncRNA, named H19, was identified among the differentially expressed lncRNAs. H19 was found as the first imprinted gene, located on human chromosome 11p15.5 H19/IGF2 gene clusters^31^. H19 plays an important role in the occurrence and development of cancer, playing the role of an oncogene in some tumors and performing the biological function of a tumor suppressor gene in some tumors^32^. The expression level of H19 has a regulatory effect on the biological functions mediated by this exosome. H19 can regulate the phenotypic effects of exosomes secreted by CD90 + cells and affect the growth of liver tumors^33^. In vitro siRNA transfection of renal cell lines inhibited the expression of the lncRNA H19, which could reduce the proliferation rate of renal cell lines and promote apoptosis^34^. LncRNA H19 also plays a role as an oncogene in non-small-cell lung cancer^35^. C-myc can directly regulate the lncRNA H19 to promote the proliferation of cancer cells and affect the prognosis of patients^36^. This experiment provides a potential biological target for molecular therapy of lung cancer^36^. Our results were consistent with a study completed by Xiong G et al., who proved that overexpression of lncRNA H19 could serve as a diagnostic marker for CAD^37^. Zhang Z et al. also found that increased plasma levels of lncRNA H19 were associated with an increased risk of CAD in a Chinese population^38^. The lncRNA H19 was also verified in the ceRNA networks. KCNQ1 overlapping transcript 1 (KCNQ1OT1) showed the highest degree of connectivity with the miRNAs, indicating its potential functions in CAD^39^. These results were also verified by Zhang Y et al., who indicated that KCNQ1OT1, HIF1A-AS2 and APOA1-AS were promising novel biomarkers for the diagnosis of CAD^40^.

Among the miRNAs in the ceRNA networks, three miRNAs, namely, miRNA-29c-3p, miR-103a-3p and miRNA-326, were regulated by H19. miRNA-29c has been proven to play important roles in gastric carcinoma, pancreatic cancer and Alzheimer's disease^41^. There is no direct evidence indicating that miRNA-29c is involved in CAD. However, the expression of miRNA-29c was identified in persistent atrial fibrillation, ischemia and cardiovascular disease, which implied the potential relevance of miR-29c in CAD^42^. As a "tumor-related microRNA", miR-103a is widely involved in the malignant biological behaviors of colorectal cancer, liver cancer, glioma and other tumors^43^. Similar to miR-29c, miR-103a has not been associated with CAD. By comparing the CAD samples with the control samples, miR-27b and miR-204 showed upregulated expression. The regulation of the expression level of the angiotensin-invertase gene has great significance in the treatment of CHD^44^. miR-27a and miR-27b were predicted to be the best candidate miRNAs for the regulation of angiotensin-converting enzymes^45^.

Seven differentially expressed mRNAs were verified in both gene datasets. The IL8 gene is a member of the chemotactic cytokine superfamily located on human chromosome 4^46^. Moreover, the combination of the IL8 gene and CXCR2 gene can increase vascular permeability^47^. However, the IL8 gene is not present in our ceRNA network, and further research may be needed to demonstrate a potential link between IL8 and CAD. CXCR2 ligands (CXCL1 and CXCL2) are small cytokines belonging to the CXC chemokine family^48^. Recent research on GWASs has found several inflammation-related loci, implicating CXCL2 in CAD risk^49^. CC chemokine ligands (CCLs) are classic cytokines that specifically regulate chemotaxis between cells^50^. De Jager et al. proved the association between CCL3 and atherosclerosis^51^. However, one limitation is these genes interactions in ceRNA networks that were predicted by GO database, lacking experimental validation. Further experimental study will verify their interactions.

## Conclusion

In this study, we successfully identified 1111 lncRNAs, 2595 mRNAs and 22 miRNAs that were differentially expressed in CAD samples. Eight genes in macrophages were also identified in the continuously up-regulated group of the time series analysis, containing HSPA1B, GPR92, TGM2, MT1JP, SPRED1, HS3ST1, MT1H and MT1M. Two genes, CD302 and CSF3R, were found down-regulated both in macrophages and time series analysis. In the ceRNA network, the lncRNAs KCNQ1OT1 and H19 showed high connectivity with the nine miRNAs. GO and KEGG results showed that genes in ceRNA networks were mainly involved in nitrogen compound metabolic process, PI3K-Akt signaling pathway and retrograde endocannabinoid signaling. These findings will improve the understanding of the occurrence and development mechanism of CAD.

## Supplementary Information

Supplementary Information 1.

## Data Availability

All data generated during the current study are available from the corresponding author on reasonable request.
