# Supplementary material for: Comprehensive analysis of the ceRNA network in coronary artery disease
Source: Sci Rep. 2021 Dec 20;11:24279. doi: 10.1038/s41598-021-03688-9 (PMC8688464; doi:10.1038/s41598-021-03688-9)

**Supplementary**

**Figure S1.** **PI3K-Akt signaling pathway.** The red rectangle represents the genes in the ceRNA network involved in this pathway. The circles represent the chemical compounds. A line with a solid arrow represents the molecular interaction or relation. Rounded rectangles represent another map. The KEGG pathway adapted, with kind permission, from Kanehisa Laboratories^[28]^.


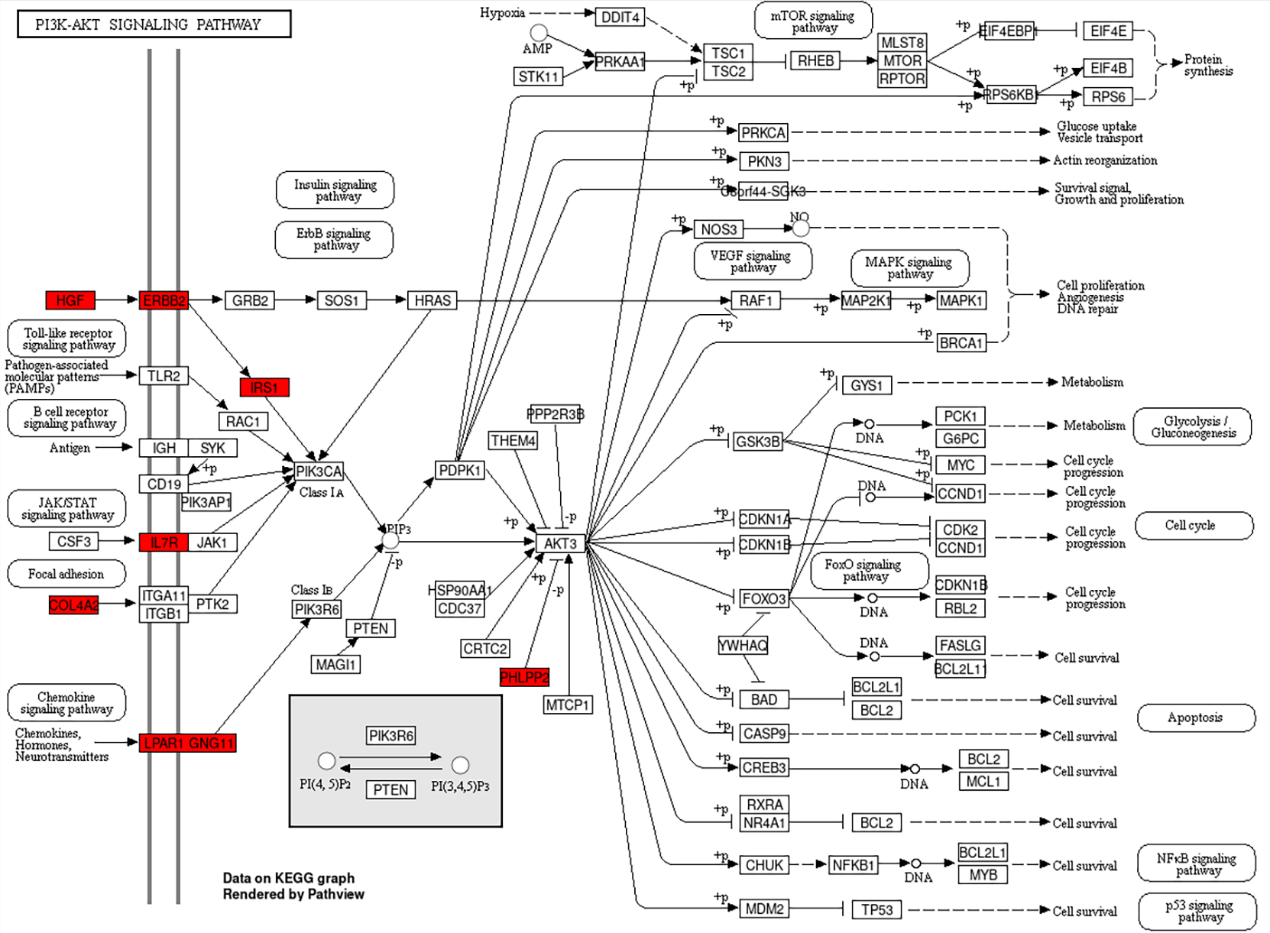

Supplement: Supplementary file 1 — Supplementary Information 1. [file 41598_2021_3688_MOESM1_ESM.docx]
